# Agreement of Doppler Ultrasound and Visual Sphygmomanometer Needle Oscillation with Invasive Blood Pressure in Anaesthetised Dogs

**DOI:** 10.3390/ani14192756

**Published:** 2024-09-24

**Authors:** Marc Armour, Joanne Michou, Imogen Schofield, Karla Borland

**Affiliations:** 1 Department of Anaesthesia and Analgesia, Lumbry Park Veterinary Specialists, Alton GU34 3HL, UK; joanne.michou@cvsvets.com (J.M.);; 2 CVS UK Ltd., CVS House, Diss IP22 4ER, UK

**Keywords:** anaesthesia, dogs, blood pressure, Doppler, invasive blood pressure, clinical study

## Abstract

**Simple Summary:**

Doppler ultrasound is a commonly utilised non-invasive blood pressure measurement technique in dogs under general anaesthesia. This prospective clinical study compared the agreement of values obtained by audible return of pulsatile flow using Doppler ultrasound and visual sphygmomanometer needle oscillation with invasive arterial blood pressure in a clinical population of anaesthetised dogs.

**Abstract:**

Visual sphygmomanometer needle oscillation (SNO) can occur before audible return of pulsatile flow (ARPF) when measuring blood pressure by Doppler ultrasound. The aim was to assess the agreement of SNO and ARPF with invasive blood pressure (iABP) in a clinical population of anaesthetised dogs. A total of 35 dogs undergoing surgery in dorsal recumbency necessitating arterial cannulation were included. Paired measurements of iABP and SNO, and iABP and ARPF, were collected. The agreement of non-invasive blood pressure (NIBP) and iABP measurements was analysed with concordance correlation coefficients (CCCs) and Bland–Altman plots. The proportions of SNO and ARPF measurements between 10 and 20 mmHg of iABP were compared. Both SNO and ARPF demonstrated greater agreement with invasive systolic (iSAP) than invasive mean (iMAP) pressures, and SNO demonstrated greater agreement with iSAP than ARPF measurements. The mean differences (95% limits of agreement) for SNO and APRF were −9.7 mmHg (−51.3–31.9) and −13.1 mmHg (−62.2–35.9), respectively. The CCC (95% CI) for SNO was 0.5 (0.36–0.64) and ARPF was 0.4 (0.26–0.54). A significantly greater proportion of SNO measurements were within 20 mmHg of iSAP compared to ARPF. Both NIBP techniques performed more poorly than veterinary consensus recommendations for device validation. Caution should be used clinically when interpreting values obtained by Doppler ultrasound in anaesthetised dogs.

## 1. Introduction

Invasive arterial blood pressure (iABP) measurement is considered the reference technique in veterinary medicine [1,2]. However, equipment, technical skill, cost, and risks involved often preclude its use in general practice, and account for its selective use in referral practice. Therefore, non-invasive blood pressure (NIBP) measurement is used in the daily anaesthetic monitoring of dogs. There is increasing availability of oscillometric devices that have demonstrated performance that is close to or meets the criteria of validation [3,4,5,6]. Despite this, Doppler ultrasound is often used.

Doppler ultrasound utilises piezoelectric crystals to transmit ultrasound waves into underlying tissues. This signal is phase-shifted when blood moves through an underlying artery and once received, the change in frequency between transmitted and reflected signals is transformed into an audible signal [7,8]. A cuff is placed proximal to the artery to be isolated and inflated until the audible signal is lost, before being slowly deflated. The cuff pressure, read by an attached sphygmomanometer, at which the first audible signal returns—or audible return of pulsatile flow (ARPF)—during deflation is taken to represent the systolic blood pressure (SAP) [7,8,9].

When ARPF is compared to the reference technique in anaesthetised dogs, the veterinary evidence base shows conflicting results, with studies reporting that Doppler ultrasound both overestimates and underestimates invasive systolic arterial blood pressure (iSAP) [5,10,11,12]. In summary, there is suboptimal precision and accuracy of Doppler ultrasound measurements and, to the best of the authors’ knowledge, this technique remains unvalidated in dogs [13]. During cuff deflation, the authors clinically noted visual sphygmomanometer needle oscillation (SNO) occurring prior to or at a similar time to ARPF. To the best of the authors’ knowledge, there is no comparison of these visual needle oscillations with invasive techniques in veterinary species. In a human study, Trigg et al. found that SNO overestimated ABP when compared to a reference technique [14].

In humans, for an NIBP device to be validated it must meet the stringent criterion set out by recognised consensus. There was a recent collaboration of internationally recognised consensus groups who have published a universal standard for the validation of such devices [15]. The American College of Veterinary Internal Medicine (ACVIM) extrapolated such guidelines to provide similar criteria for NIBP devices in veterinary medicine, against which these devices are compared for use in small animals [2,16].

The aim of this study was to compare the agreement of ARPF and SNO with iABP in a clinical population of anaesthetised dogs. We hypothesised that SNO would demonstrate greater agreement with iSAP when compared to ARPF.

## 2. Materials and Methods

This prospective, clinical study was performed at a private, small animal referral hospital in the United Kingdom. Ethical approval was granted by the institution’s internal ethical review committee (CVS-2022-014). Informed owner consent was obtained.

### 2.1. Animals

A sample size calculation which assumed a mean difference of 10 mmHg in paired blood pressure measurements, with 15 mmHg standard deviation, revealed that 36 dogs were to be recruited to gain 80% power at a 95% confidence level [2,17]. Dogs were included if they were anaesthetised for a surgical procedure which necessitated arterial cannulation and if they were positioned in dorsal recumbency. Dogs were excluded if they were positioned in an alternate recumbency, there was no demonstrable consent, there was inability to cannulate a dorsal pedal artery, there were too few clinical staff to facilitate data collection, or there was marked anaesthetic instability (for example, but not limited to, haemodynamically significant arrhythmia, obvious pulse pressure variation, and impending cardiopulmonary arrest) deemed by a resident or clinician in anaesthesia and analgesia. Anaesthetic management of individual cases was dictated and supervised by a European specialist in anaesthesia and analgesia.

### 2.2. Instrumentation

A dorsal pedal artery was aseptically prepared and cannulated with a 20- or 22-gauge catheter (Jelco^®^ IV catheter 20 g and 22 g, Smiths Medical ASD, Inc., Minneapolis, MN, USA) and connected to a single lumen T-port (T-port extension, B Braun Vet Care GmbH, Tuttlingen, Germany).

The contralateral limb circumference at the level proximal to the hock was measured and a new blood pressure (BP) cuff, with a width closest to 40% of limb circumference [18], was applied (SoftCheck^®^ blood pressure cuff, vinyl neonatal #1-5 double tube, Statcorp Medical, Snoqualmie, DC, USA). The plantar aspect of the metatarsus was clipped and ultrasound coupling gel was applied (Vue™ ultrasound gel, 250 mL, Optimum Medical Solutions Ltd., Leeds, UK).

A 500 mL bag of 0.9% Sodium Chloride (Sodium Chloride 0.9 g/100 mL, 500 mL, B. Braun Vet Care, Melsungen, Germany) was heparinised to 5 IU/mL (Heparin Sodium, 1000 IU/mL, 5 mL, Wockhardt UK Ltd., Wrexham, UK). This was connected to an invasive pressure monitor kit with non-compliant tubing that was replaced between each animal (LogiCal^®^ Single pressure monitoring kit, 180 cm plus 30 cm extension, Smiths Medical ASD, Inc., Minneapolis, MN, USA). The system was primed as per manufacturer instructions and the dome attached to the transducer. The fluid bag was pressurised to 300 mmHg and the system flushed to remove bubbles. The system was zeroed to atmospheric pressure and the transducer placed at the level of the manubrium. The monitoring kit tubing was connected to the arterial catheter via the T-port and the iABP was displayed numerically and graphically via the multiparameter monitor (Datex Ohmeda S/5 anaesthesia monitor, Datex-Ohmeda, Helsinki, Finland).

Ultrasound coupling gel was applied to the Doppler transducer (Ultra Vet 8.2 Doppler flow detector with infant flat probe 8.2 MHz, Parks Medical Electronic Inc., Aloha, OR 97078, USA), which was positioned on the prepared plantar metatarsus and secured with Durapore tape (3M™ Durapore™ medical tape, 3M PLC, Bracknell, UK). The Doppler was turned on to ensure the pulse was audible and adjusted until such. All the equipment was prepared and applied by a single author.

Prior to data collection, the sphygmomanometer (Veterinary Welch Allyn shock resistant DS-65 sphygmomanometer, Welch Allyn Inc., Skaneateles Fall, NY, USA) was calibrated to manufacturer standards using a digital pressure manometer (Digitron™ 2025P Absolute Pressure Meter, Digitron, Wales, UK). Multiparameter monitors were routinely serviced and calibrated to manufacturer standards.

### 2.3. Data Collection

**Figure 1:**
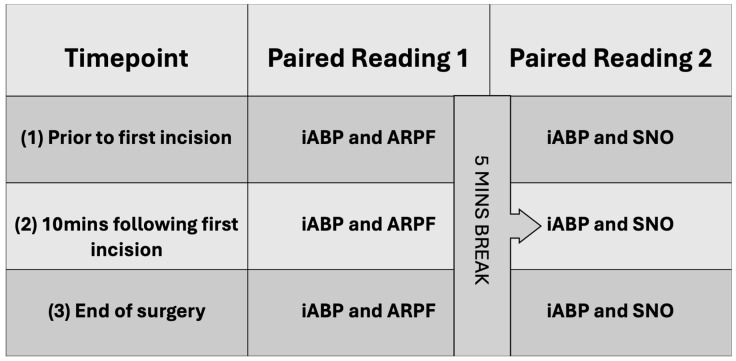
Overview of data collection.

A single person was responsible for the NIBP measurements and was blinded to the iABP values. A second person was responsible for iABP recording and was blinded to the NIBP values. A third person was responsible for monitoring the anaesthetic. Three sets of paired readings were performed: prior to first incision; 10 min following first incision; and at the end of surgery. Each set of readings included one iABP measurement paired with the average of five ARPF measurements. Five minutes elapsed and then another iABP measurement was repeated and paired with the average of five SNO measurements (Figure 1).

An iABP measurement was taken following a subjective fast flush test to assess system damping; once the arterial trace stabilised, the iSAP and iMAP were recorded. Person two then observed the iABP trace whilst NIBP readings were completed. If either value changed by 10 mmHg or more, the paired reading was discarded and repeated. An ARPF reading was taken by inflating the cuff until no audible pulse was present; then, pressure was slowly released from the sphygmomanometer until a sound was audible; the corresponding pressure read at the time of the first audible sound was recorded. An SNO reading was taken by inflating the cuff to a suprasystolic value before slowly releasing pressure from the sphygmomanometer. The corresponding value on the sphygmomanometer at which an oscillation was first visualised was recorded. The NIBP techniques were repeated five times and averaged for each paired reading; neither technique was timed.

Readings were taken at a subjectively stable plane of anaesthesia; the effect(s) of any intervention (e.g., controlled mechanical ventilation, new drug, or drug infusion) performed between timepoints was allowed to plateau before the new paired readings were taken, such that each paired reading was taken under a closely matched anaesthetic plane and condition.

### 2.4. Statistical Analysis

Data were assessed for normality using Shapiro–Wilk tests and visual inspection of histograms. Normally distributed data were summarised using the mean and standard deviation. The reliability of the five NIBP measurements that attributed to the mean measurements were compared by intraclass correlation coefficients (ICCs). The agreement of the iSAP, iMAP, and NIBP measurements (ARPF and SNO) were analysed with concordance correlation coefficients (CCCs) and Bland–Altman plots [19,20]. Coefficients of 1 demonstrate perfect agreement; those of 0 indicate no agreement. Coefficients (ICCs and CCCs) were interpreted as >0.8 strong agreement, >0.6–0.8 moderate agreement, >0.3–0.6 fair agreement, and >0.1–0.3 poor agreement [21]. Mean differences between the iABP and NIBP techniques were calculated to obtain the bias. The standard deviation (SD) and 95% limits of agreement (mean ± 1.96SD) were also calculated and graphically plotted. Mean differences (bias) from the Bland–Altman that are closest to 0 indicate greater agreement. The proportion (and corresponding confidence intervals) of NIBP measurements falling within 10 and 20 mmHg of the iABP measurement were reported. The difference between the ARPF and SNO proportions were compared using two sample Z-tests. Statistical analysis was performed using the software Stata 17.0 (Stata, TX, USA). A *p*-Value < 0.05 was considered statistically significant.

## 3. Results

### 3.1. Population

The mean age was 7.5 years (SD 3.6; range 0.5–12.9 years). There were 21 male (60%) and 14 female dogs (40%). The mean bodyweight was 19.6 kg (SD 10.3; range 4.8–40 kg), with a mean body condition score of 5.2/9 (SD 1.4). The majority of cases were assigned American Society of Anaesthesiologists Physical Status Classification scores of III (*n* = 22; 62.86%) and II (*n* = 9; 25.71%) [22,23]. The remaining dogs were assigned scores of IIIE (*n* = 2; 5.71%), IV (*n* = 1; 2.86%), and IVE *(n* = 1; 2.86%). Anaesthetic management was similar for the majority of subjects and is summarised in Table 1. The surgical procedures included extrahepatic portosystemic shunt attenuations, laparoscopic spay, laparoscopic liver biopsies, cervical vertebral surgeries, mass removals of the neck and axilla, ventral neck explore and foreign body removal, abdominal lymph node extirpations, percutaneous coil embolisation of intrahepatic portosystemic shunts, cholecystectomy, gastrostomy, and nephrectomy.

A total of two dogs were excluded due to anaesthetic instability. A total of 35 dogs were included in the statistical analysis. For these 35 dogs, a total of 16 paired readings were excluded from data analysis due to absence of SNO (*n* = 5), anomalous result (*n* = 1), or anaesthetic complication (*n* = 10, including marked pulse pressure variation, a peri-arrest period after inadvertent drug error, hypotension requiring more than one vasopressor ± blood product, and a subject being moved from theatre before timepoint three reading was possible). Subsequently, a total of 98 paired readings for ARPF were compared with a total of 96 paired readings for SNO. A total of 194 iSAP readings were included in analysis; of these, four (2.1%) iSAP readings were subjectively underdamped and 32 (6.1%) were subjectively overdamped. NIBP recordings were not timed; subjectively, SNO readings took longer on average to record where weak or subtle oscillations were observable compared to ARPF (where reliable audible signal was present).

### 3.2. Measurements

**Figure 2:**
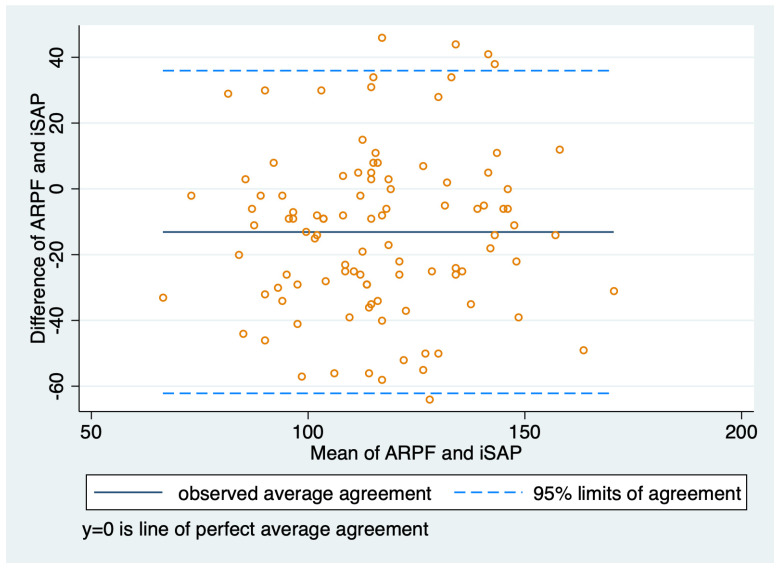
Bland–Altman plot for agreement between invasive systolic arterial pressure and audible return of pulsatile flow measurements.

**Figure 3:**
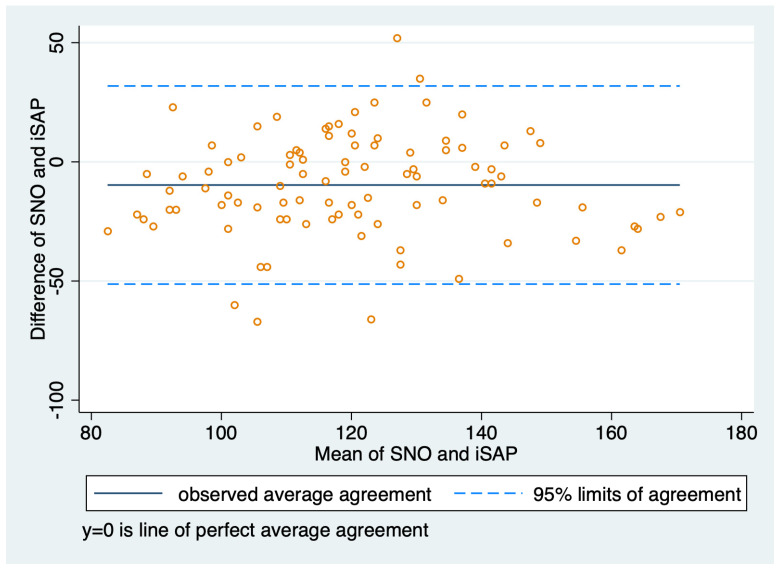
Bland–Altman plot for agreement between invasive systolic arterial pressure and sphygmomanometer needle oscillation measurements.

All blood pressure measurements were normally distributed. For paired measurements of iABP and ARPF, the mean iSAP was 122.9 mmHg (SD 23.60; range 67–188 mmHg), the mean iMAP was 73.1 mmHg (SD 14.50; range 44–133 mmHg), and the mean ARPF was 108.7 mmHg (SD 25.75; range 34–164 mmHg). For paired measurements of iABP and SNO, the mean iSAP was 124.2 mmHg (SD 22.32; range 81–181 mHg), the mean iMAP was 74.3 mmHg (SD 13.14; range 46–121 mmHg), and the mean SNO was 115.5 mmHg (SD 22.64, range: 68.0–160.0 mmHg). A performance summary of both NIBP techniques is shown in Table 2 and Table 3; Bland-Altman plots were also compared (Figure 2 and Figure 3). A total of 21 (10.8%) paired readings were performed during hypotension (iMAP < 60 mmHg), there were two readings during hypertension (iMAP > 120 mmHg), and 171 paired readings were performed during normotension (iMAP 60–120 mmHg). The ICC of ARPF measurements was 0.99 (95% CI, 0.98–0.99) and the ICC of SNO measurements was 0.97 (95% CI, 0.96–0.98), indicating a more reliable performance of ARPF compared to SNO and more variation within the five SNO readings before being averaged.

## 4. Discussion

The aim of this study was to compare NIBP readings obtained by visual SNO and ARPF (using Doppler ultrasound) with iABP readings in a clinical population of anaesthetised dogs. SNO agreed more closely with iSAP when compared to ARPF, evidenced by a smaller mean difference and closer limits of agreement; however, the difference was small overall. ARPF agreed more closely with iSAP when compared to iMAP, in agreement with previous studies [5,10,11,12]. This study found that both ARPF and SNO tended to underestimate iSAP (Table 2). The 95% LOAs for NIBP techniques were very wide, indicating poor precision. This study did not seek to validate either technique, but to merely compare their performance in a clinical setting. Our study adds weight to the existing literature and helps to inform clinical practice by demonstrating that Doppler ultrasound may be utilised for trends, but iABP or a validated NIBP device is preferred if specific targets of BP values are to be met.

This study found negative biases of −13.1 and −9.7 mmHg for ARPF and SNO, respectively, when compared to iABP, in alignment with Vachon et al. (2014) (−4.1 mmHg) and Garofolo et al. (2012) (−6 mmHg), and contrary to Seliškar et al. (2013) (27 mmHg) and Bourazak and Hofmeister (2018) (2.8 mmHg) [5,10,11,12]. All aforementioned studies investigating the use of Doppler ultrasound in anaesthetised dogs found very wide LOAs, in agreement with our study, indicating the poor precision of the technique. Not all studies presented proportions of Doppler measurements falling between 10 and 20 mmHg of iABP; those that did generally showed higher proportions than this study.

Comparison with results of other studies is limited by differences in instrumentation, population, procedure, study methods, and differing cut-offs for hypo-, hyper-, and normotension. It has been shown that agreement of iABP and ARPF measurements can be affected by the location of both pieces of equipment. Cerejo et al. (2020) found that BP cuffs above the hock and distal 1/3rd antebrachium had greater agreement with iABP measured from the dorsal pedal artery in cats, suggesting agreement may change between studies depending on the site of BP cuff and the artery cannulated [24]. Doppler readings can also be affected by poor BP cuff condition, poor BP cuff size selection, position of the cuff in relation to the heart, limb movement, user experience, and inter-user variation [25]. Moreover, both Moll et al. (2018) and Kennedy and Bartella (2015) demonstrated poor agreement and overestimation of Doppler measurements in anaesthetised dogs <5 kg, indicating patient size may also affect performance [26,27]. In this study, we used a new cuff for each patient at the same anatomic site, measured the limb circumference with a tape measure to choose an appropriate size cuff, and had a single person take the NIBP readings to reduce further sources of variation and error. Only one dog included weighed <5 kg, so the small size of subjects was unlikely to skew results. Five consecutive NIBP readings were averaged and compared to iABP to increase the precision (i.e., narrow the LOA) of ARPF measurements, as recommended by ACVIM guidelines [16].

Invasive BP readings may be affected by the level of the transducer with respect to the heart, contact of the cannula with the arterial wall, and the presence of blood clots and air bubbles, resulting in overdamping and underestimation of iSAP [1,7]. In our study, the iABP monitoring kit was set up as per the manufacturer’s instructions, which limits the formation of microbubbles in the saline column, and a pressurised bag of heparinised saline was used to reduce clot formation. Furthermore, a fast flush test was performed prior to each reading to assess system damping and hence assess the reliability of the readings. In our study, 91.8% of iABP readings were under subjectively normal conditions; the degree of error introduced by the inclusion of 8.2% (*n* = 6) of iABP readings over- or underdamped in the study remains unclear. Attempts were made to improve system conditions, but the elimination of all variation was not possible due to the clinical nature of the study. It is suggested that both the use of vasoactive drugs and arterial cannula size may impact iABP readings, but this has never been quantified in the literature [28]. This study made reasonable adjustments to reduce sources of preventable error and variation.

Limitations associated with instrumentation are inherent and include an inability to use the same limb for NIBP and iABP concurrently and temporal disparity to complete simultaneous readings. This study attempted to account for this by using unobstructed contralateral pelvic limbs at the same height in dorsal recumbency, such that the distance and height from the heart should have been equal and by ensuring any iABP measurement varying by 10 mmHg or more was discarded and the paired reading repeated at a more stable plane of anaesthesia.

During data collection, the bias of individual data collectors was minimised by blinding them to each other’s readings and with the inclusion of a washout period. However, the order of NIBP data collection was not randomised, and increasing proportions of ARPF measurements between 10 and 20 mmHg of iSAP over the three paired measurement timepoints could indicate measurement bias. Future studies should aim to standardise instrumentation and methods to allow for more direct comparison when investigating new devices and publishing results in similar formats.

Trigg et al. (2019) investigated the ‘visual needle jump’ of sphygmomanometers in conscious human subjects and found it overestimated a reference technique by 14–15 mmHg, concluding it can only be used as an estimation of BP [14]. This contrasts the finding of this study, where SNO tended to underestimate the reference technique. In their discussion, the authors speculate that the needle oscillations are related to pressure changes transmitted through the BP cuff in relation to the pulsatile increase in the volume of the artery as the BP cuff is deflated [14]. To the authors’ knowledge, such needle jumps or oscillations have not been described in the veterinary literature.

In this study, the SNO technique failed to record values in five paired readings, compared with no failed readings for the ARPF (Doppler) technique. One ARPF reading was unsuccessful; giving markedly low readings compared with iABP values. This could have been due to BP cuff slip, equipment error, or a true error. There was a subjective variation in the observed strength of needle oscillation during data collection, leading to differences in the time taken to obtain readings. Potentially, weak oscillations could have been visually missed and are picked up by automated oscillometric systems more accurately. Finally, we speculate that inter-user variations using SNO could be high, and the potential for failed readings with this technique may preclude its clinical use. Hence, further research is needed before any results obtained by SNO can be used clinically or interpreted with weight.

A limitation of the study includes the use of a clinical population that was heterogeneous; controlling the anaesthetic protocol, anaesthetic management, and surgical procedure (i.e., level of sympathetic stimulation) would have allowed closer comparison between patients included in this study but would not have represented a true clinical population for which these technologies are used daily. Moreover, each patient effectively served as their own control, with test conditions that were close to the same when each paired reading was evaluated. Further research, including inter-user variability in more controlled conditions could be used to further evaluate the performance of SNO’s utility for NIBP measurement in a clinical setting.

## 5. Conclusions

This study found that SNO more closely agreed with iSAP when compared to ARPF. This suggests that when performing Doppler ultrasound blood pressure measurement in anaesthetised dogs, if SNO occurs preceding ARPF it may represent iSAP more closely. Overall, both NIBP techniques performed poorly, especially when compared to published results for alternate NIBP techniques utilising oscillometry. Where maintenance of ABP within narrow margins is of critical importance, iABP measurement or a validated non-invasive measurement device should be used in preference of Doppler ultrasound.

## Figures and Tables

**Table 1:** Overview of medications used for anaesthetic management of included subjects. Numbers (*n*) represent inclusion of drug use for individual patient protocol for each the 35 dogs included in the study.

Premedication	Induction	Maintenance	Adjunctive Infusion(s)	Blood Pressure Support—Infusion	Blood Pressure Support—Bolus
α-2 Agonist + Opioid (30)	Propofol or Alfaxalone (15)	Volatile anaesthetic (34)	Fentanyl (10)	Dopamine (7)	Glycopyrrolate (3)
α-2 Agonist (2)	Propofol or Alfaxalone and Midazolam (10)	Total Intravenous Anaesthesia: Propofol (1)	Dexmedetomidine (2)	Noradrenaline (6)	Ephedrine (4)
Acepromazine + Opioid + α-2 Agonist (1)	Propofol or Alfaxalone and Ketamine (7)		Ketamine and Fentanyl (4)	Dobutamine (2)	
Opioid (2)	Propofol or Alfaxalone and Fentanyl (1)		Lidocaine (1)		
	Alfaxalone or Propofol and Midazolam and Fentanyl (2)		Ketamine (6)		
			Fentanyl and Dexmedetomidine (2)		
			Fentanyl & Ketamine and Dexmedetomidine (1)		

**Table 2:** Comparison of invasive systolic arterial blood pressure (iSAP) measurements with corresponding paired audible return of pulsatile flow (ARPF) blood pressure measurements. Results reported include concordance correlation coefficients (CCC) and their 95% confidence intervals (CI), the mean difference between readings and their 95% limits of agreement (LOAs), and the proportion (%) of ARPF readings within 10 and 20 mmHg of iSAP.

Agreement	CCC (95% CI)	Mean Difference mmHg (95% LOAs)	ARPF Readings < 10 mmHg of iSAP (95% CI)	SNO Readings < 20 mmHg of iSAP (95% CI)
ARPF vs. iSAP (Average)	0.40 (0.26–0.54)	−13.1 (−62.2–35.9)	37.5% (28.6%–47.3%)	54.8% (45.1%–64.2%)
ARPF vs. iSAP (timepoint 1)	0.39 (0.14–0.64)	−18.4 (−77.4–40.7)	31.4% (18.2%–48.5%)	45.7% (30.1%–62.3%)
ARPF vs. iSAP (timepoint 2)	0.37 (0.11–0.64)	−12.0 (−70.8–46.8)	37.1% (22.8%–54.2%)	57.1% (40.4–72.4)
ARPF vs. iSAP (timepoint 3)	0.41 (0.15–0.67)	−12.1 (−54.2–30.0)	44.1% (28.5%–61.0%)	61.8% (44.5–76.4)

**Table 3:** Comparison of invasive systolic arterial blood pressure (iSAP) measurements with corresponding paired visual sphygmomanometer needle oscillation (SNO) blood pressure measurements. Results reported include concordance correlation coefficients (CCC) and their 95% confidence intervals (CIs), the mean difference between readings and their 95% limits of agreement (LOAs), and the proportion (%) of ARPF readings within 10 and 20 mmHg of iSAP.

Agreement	CCC (95% CI)	Mean Difference mmHg (95% LOAs)	SNO Readings < 10 mmHg of iSAP (95% CI)	SNO Readings < 20 mmHg of iSAP (95% CI)
SNO vs. iSAP (Average)	0.50 (0.36–0.64)	−9.7 (−51.3–31.9)	38.3% (28.9–48.6)	76.6% (66.9–84.1)
SNO vs. iSAP (timepoint 1)	0.54 (0.30–0.78)	−6.7 (−46.9–33.5)	39.4% (24.3–56.9)	72.7% (55.1–85.3)
SNO vs. iSAP (timepoint 2)	0.50 (0.27–0.74)	−13.2 (−56.2–29.8)	40.6% (25.1–58.3)	75.0% (57.1–87.1)
SNO vs. iSAP (timepoint 3)	0.45 (0.17–0.72)	−9.3 (−51.1–32.5)	34.5% (19.5–53.3)	82.8 (64.4–92.7)

## Data Availability

The data presented here are available upon request from the corresponding author.
